# Declining trend in anemia prevalence among non-pregnant women of reproductive age in Vietnam over two decades: A systematic review and meta-analysis of population studies

**DOI:** 10.34172/hpp.2022.30

**Published:** 2022-12-10

**Authors:** Nguyen Trung Kien, Tran Quang Duc, Vu Thi Quynh Chi, Phan Ngoc Quang, Bui Thi Thanh Tuyen, Dinh Thi Phuong Hoa

**Affiliations:** ^1^Department of Obstetrics and Gynecology, Thai Binh University of Medicine and Pharmacy, Thai Binh, 06121, Vietnam; ^2^Independent Scholar, Tokushima, Japan; ^3^Nutrition Department, Dong A University, Danang, 50000, Vietnam; ^4^The Center Service for Technology Science of Medi-Phar, Thai Binh University of Medicine and Pharmacy, Thai Binh, 06121, Vietnam; ^5^Faculty of Health and Environmental Sciences, Auckland University of Technology, 1010, Auckland

**Keywords:** Vietnam, Women, Anemia, Anaemia, Non-pregnancy

## Abstract

**Background:** Anaemia is a public health concern in developing nations, particularly among women of reproductive age. However, the present prevalence and recent trend in anaemia among this population are unclear. This systematic review aimed to evaluate the prevalence of anaemia among non-pregnant women in Vietnam.

**Methods:** We systematically searched databases such as PubMed, Scopus, and reference lists of earlier prevalence studies from their inception until July 2022. For statistical analysis to check for heterogeneity, random or fixed effects models were employed to summarize the prevalence of anaemia. Visual examination of a funnel plot was used to determine the presence of publication bias, which was then verified using the Egger regression test. Subgroup analyses were also undertaken to evaluate how the proportion of anaemia differs across various study groups.

**Results:** A total of 188 studies were found as a result of the bibliographical search. Finally, of the 12 included studies, anaemia affected 5089 non-pregnant women out of a total of 19744, making the prevalence of this condition 23.2% (95% CI: 16.1-32.2). From 1995 to 2013, the prevalence of anaemia in this population declined significantly, from 42.6% to 16.9%. Notably, the prevalence of anaemia among non-pregnant women differed by geography and increased by mountains, Northern Vietnam, rural areas, and ethnic minority groups. Furthermore, no publication bias was found in this meta-analysis.

**Conclusion:** To enhance the health of women and meet global objectives for eliminating anaemia, more efforts are required in specific regions and ethnic minority groups in Vietnam.

## Introduction

 In women, anaemia compromises health and well-being and raises the likelihood of unfavourable maternal and neonatal outcomes.^1^ In 2019, the prevalence of anaemia in women of reproductive age was 29.9% (95% UI: 27.0-32.8), which is comparable to more than half a billion women aged 15-49 years worldwide.^2^ Anaemia is a problem that affects the public health of developing nations, particularly among women and children. Anaemia has been linked to higher mortality and morbidity in women, stunted development in children, cognitive decline, an increased risk of infection of many sorts, and decreased productivity at work, all of which have a significant financial impact on families and the whole society.^3,4^

 Despite notable gains in socioeconomic and health status in the majority of low-income Southeast Asian nations, these countries continue to face challenges in lowering a high burden of malnutrition, which contributes to the high percentage of anaemia among non-pregnant women.^5^ The percentage of people who suffer from anaemia is on the decrease, falling from an estimated 39.8% in 1990 to an estimated 37.5% in 2010.^6^ Despite this, progress has been significantly less than predicted in lowering the percentage of anaemia, and its socio-economic impact, especially in resource-poor nations, remains a significant concern.^6^ In light of the slow progress that has been made in reducing the percentage of anaemia, the World Health Assembly in 2012 adopted a goal of a decrease in anaemia prevalence of 50% among women of reproductive age by the year 2025.^1,7^ According to the worldwide prevalence of 29%–38% anaemia among women of reproductive age in 2011, achieving this aim would need a drop of 1.8%–2.4% points each year among this population.^8^

 Vietnam, with a population of 98.51 million people and among those have 24.7 million women at reproductive age (15-49), is a developing nation in Southeast Asia.^9^ The reduction of anaemia in women of reproductive age is of critical importance to the protection and promotion of public health. This kind of information is required to identify the social and economic burden of anaemia and maximize the use of available medical resources to enhance anaemia’s diagnosis, treatment, and prevention. In Vietnam, several population-based studies representative of a single region/province have been carried out throughout the last two decades; nevertheless, there is no estimate of the prevalence over the whole nation or of the trend during this period. Therefore, we performed a systematic literature review and meta-analysis to provide a comprehensive and up-to-date evaluation of the prevalence of anaemia among non-pregnant women in Vietnam.

## Material and Methods

 This systematic review and meta-analysis project was carried out in accordance with the protocol that had been published and submitted to the International Prospective Register of Systematic Reviews. The Preferred Reporting Items for Systematic Reviews and Meta-Analyses (PRISMA) standards were adhered to throughout this meta-analysis and systematic review.^10^

###  Search procedure

 A comprehensive search of the scholarly literature was carried out in order to locate prospective publications that had been published on the frequency of anaemia among the population of reproductive women in Vietnam. PubMed and Scopus databases were carefully searched in July 06, 2022 with no time constraint. A list of search techniques was compiled by the search team, which included the following keywords: “Vietnam”, “Vietnamese”, “Women”, “Adult”, “Non-pregnancy”, “Anemia”, “Anaemia”, “Haemoglobin”, “Hemoglobin”, “Haematocrit”, “Hematocrit”. After settling on a list of pertinent keywords, database searches were conducted using those keywords, using the “AND” and “OR” operators chained together as necessary. The following PubMed and Scopus advanced search options were employed: “(Vietnam[Title/Abstract] OR Vietnamese[Title/Abstract]) AND (Women[Title/Abstract] OR Adult[Title/Abstract] OR non-pregnancy[Title/Abstract]) AND (anemia[Title/Abstract] OR anaemia[Title/Abstract] OR haemoglobin[Title/Abstract] OR hemoglobin[Title/Abstract] OR haematocrit[Title/Abstract] OR hematocrit[Title/Abstract])”, and “TITLE ((vietnam OR vietnamese) AND (women OR adult OR non-pregnancy) AND (anemia OR anaemia OR haemoglobin OR hemoglobin OR haematocrit OR hematocrit)) TITLE-ABS-KEY (((vietnam OR vietnamese ) AND (women OR adult OR non-pregnancy) AND (anemia OR anaemia OR haemoglobin OR hemoglobin OR haematocrit OR hematocrit))) respectively. There were no linguistic limitations. In addition, we combed through the reference lists of significant publications to identify possibly relevant other articles.

###  Selection of studies

 Using the inclusion criteria, two separate researchers evaluated the eligibility of all abstracts retrieved from our searches after a preliminary assessment of the titles acquired. In the case of dispute among reviewers, a third reviewer evaluated the research and a decision for inclusion were made by unanimous consent.

###  Inclusion criteria

 The following criteria were used for inclusion:

- Population: the research involved non-pregnant women of reproductive age (15-49 years).
- Study area: The study was only conducted in Vietnam.
- Study design: The article must fulfill all of the following prerequisites for inclusion: (1) was an original study or national survey; (2) was able to determine the prevalence of anaemia for the non-pregnant women of reproductive age; and (3) comprised a survey of either the general population or the community at large.
- Publication condition: the article (1) was written in English, and (2) was published in a peer-reviewed journal.

###  Exclusion criteria

 The studies were disqualified based on the criteria listed below:

- Research on the prevalence of anaemia in certain groups, such as those suffering from cancer, HIV/AIDS, dengue, renal disease, diabetes, and pregnant and postpartum women.
- Animal-based research.
- Review articles, case studies, letters, commentaries, protocols, conference abstracts and case-control studies.
- Methodologically flawed articles.
- Duplicated studies or duplicate populations from another research included in the review. Only one of these studies had the most significant population sample to be included in our overall prevalence estimate.

###  Data extraction

 Data was gathered from a selection of articles by three separate authors (TQD, BTTT and DTPH) based on criteria that were previously established. These researchers then went through each full-text study to determine its eligibility. For each study found to be eligible, data were extracted separately using Microsoft Excel 2019 for macOS. The following information was gleaned from the retrieved full-text article: the full name of the first author and year of publication, study design and period, sample size, geographic (mountain/plain), areas (urban/rural), regions (Northern/Central/Southern Vietnam), partial or complete ethnic minority participation (yes/no) and percentage of participants with anaemia. All authors participated in a conversation to resolve any discrepancies in the data extraction procedure.

###  Qualitative evaluation of studies

 Using an adapted version of the Newcastle-Ottawa Scale that was designed specifically for use in cross-sectional and case-control studies,^11^ three authors (NTK, TQD and PNQ) independently evaluated the degree of methodological rigor present in each of the included studies. This standardized tool includes three primary indications pertaining to the methodological characteristics, the comparability, and the statistical analysis of publications. According to the list of questions provided for each indication, the authors must choose the most appropriate answer for the included article in question. A score was only given to an answer that demonstrated high quality. We divided the overall quality of each included study into four categories, as follows: very good studies (8 to 9 points), good studies (6 to 7 points), satisfactory studies (4 to 5 points) and unsatisfactory studies (0 to 3 points). The final analysis did not include any unsatisfactory studies. The online appendix file may include details on how the final scores were calculated.

###  Statistical analysis

 After the data extraction process was completed using the Microsoft Excel program, the data were then exported to the RStudio Desktop 2022.07.0 + 548 version software for macOS 10.15 + (https://posit.co/download/rstudio-desktop/). The meta-analyses were conducted using ‘meta’, ‘metafor’, and ‘ggplot2’ packages. The I^2^ statistic was used to determine the degree of heterogeneity between included studies.^12^ It was anticipated that the I^2^ would be high, and the prevalence of anaemia could dissimilarity between studies due to genuine differences in the characteristics of included studies and the potential factors (e.g. various collecting periods, socio-economic circumstances, and dietary habits in different geographic regions) that might affect the anaemia condition of Vietnamese non-pregnant women of reproductive age. Consequently, viewed from these angles, we estimated the pooled prevalence using a random-effects model with a 95% confidence interval (CI). Subgroup analyses were performed by period, design, sampling, geographics, areas, regions and ethnic minority participation in the included studies. To investigate publication bias, we analyzed the symmetry of the funnel plots and used the Egger’s regression test; *P* < 0.10 was judged statistically significant. We also did a sensitivity analysis, in which we excluded one research at a time, to elucidate the impact of individual studies on the overall pooled estimate and to determine influential studies.

## Results

**Figure 1:**
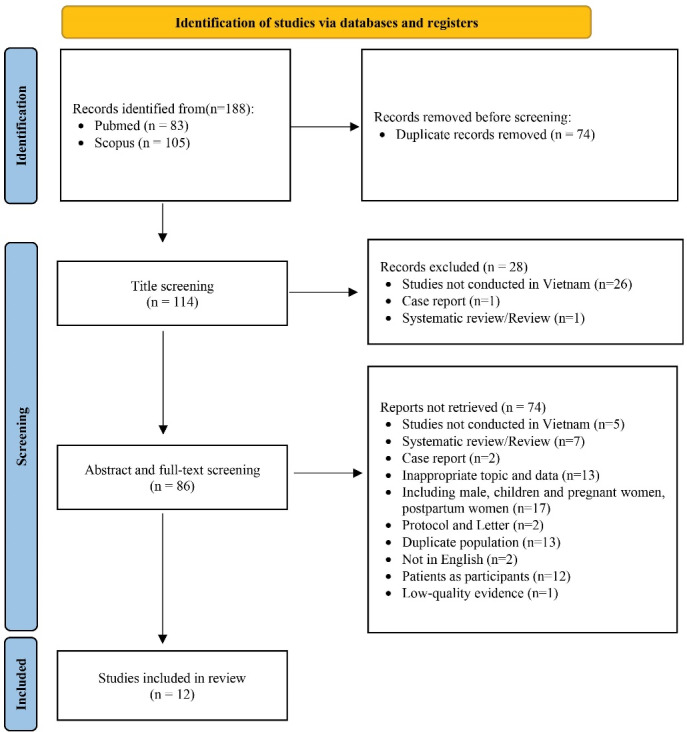
A graphical depiction of the search for relevant literature to include in the meta-analysis

Figure 1 presents a summary of the number of studies that were found, reviewed, and ultimately included in our research using the PRISMA flow diagram. The initial literature search yielded a total of 188 possible articles. Out of these, 74 duplicate articles were eliminated; 28 of them were then omitted from further analysis because, based on their titles, they did not fit the inclusion requirements. Thus, only 86 research papers were selected for abstract and full-text evaluation. The graphical representation of the textual explanations for retrieved but not included articles were displayed. Of the 74 excluded publications reviewed, one^13^ was discarded because it did not give sufficient information regarding sample size, participant selection, and comparisons between groups, even though fulfilling the requirements for inclusion. Following an in-depth evaluation, the quantitative synthesis includes a total of 12 different papers that were published between the years 2003 and 2017.^14-25^

###  Included study characteristics

Table 1 shows the characteristics of the included studies. There were a total of 19 744 women of reproductive age who had their anaemia checked with the number of participants in each research varies anywhere from 72^15^ to 6774,^20^ and the data from the included researches were collected from 1995^20^ to 2013.^18^ The majority of research used cross-sectional designs and random sampling as their primary data collection methods. Most of the research was done in two regions of Vietnam. Among them are one research from the Central Vietnam region,^16^ nine studies from the Northern Vietnam region,^14,15,17-19,21,23-25^ one national study,^20^ and one study on a large-scale.^22^ Additionally, there were six studies from plain areas,^14,15,17,21,23,25^ four studies from mountain areas,^16,18,19,24^ and one study from both plain and mountain areas.^22^ Haemoglobin (Hb) values below 12.0 g/dL or 120 g/L were utilized for all included studies as the diagnostic threshold for anaemia among non-pregnant women of reproductive-aged 15–49 years old. It has been observed that the prevalence of anaemia in non-pregnant women ranges from 9.3%^17^ to 45.6%,^25^ with the greatest and lowest values correspondingly. Only one of the articles has obtained the minimum score required for inclusion on the quality evaluation scale^15^; the other articles all have scores of 8 or above. The score of each included article is shown in Table 1.

**Table 1:** Characteristic of studies on the prevalence of anaemia among Vietnam non-pregnant women of reproductive age

**Authors**	**Period**	**Design**	**Total**	**Sampling**	**Regions**	**Rural** **/Urban**	**Mountain** **/Plain**	**Ethnic minority**	**Anaemia %**	**Define**	**Score**
Van Thuy et al,^14^ 2005	2000-2005	Case-control study	576	Random	Northern Vietnam	Rural	Plains	No	24.5	Hb ^a^ < 120 g/L	9
Van Nhien et al,^15^ 2006	2000-2005	A cross-sectional study	72	Non-random	Northern Vietnam	Rural	Plains	No	38	Hb < 120 g/L^b^	5
Trinh and Dibley, 2007^18^	2000-2005	A cross-sectional study	272	Random	Central Vietnam	Rural	Mountains	Yes	19.9	8-11.9 g/dL^c^	9
Van Thuy et al,^19^ 2003	2000-2005	A cross-sectional study	2159	Random	Northern Vietnam	Urban	Plains	No	9.3	Hb < 120 g/L	9
Ramakrishnan et al,^20^ 2016	≥ 2006	Case-control study	5011	Random	Northern Vietnam	Rural	Mountains	Yes	20	Hb < 12 g/dL	9
Pasricha et al,^21^ 2009	≥ 2006	A cross-sectional study	221	Random	Northern Vietnam	Rural	Mountains	No	36.7	Hb < 120g/L	8
Nguyen et al,^22^ 2006	≤ 1999	A cross-sectional study	6774	Random	National Study	National Study	National Study	National Study	39.9	Hb < 12 g/dL	9
Nakamori et al,^23^ 2010	≥ 2006	A cross-sectional study	1759	Random	Northern Vietnam	Urban	Plains	No	9.6	Hb < 120 g/L	8
Laillou et al,^24^ 2014	≥ 2006	A cross-sectional study	1530	Random	19 Provinces	Both	Both	Yes	11.2	Hb < 120 g/L	8
Hall et al,^25^ 2017	≥ 2006	Case-control study	117	Random	Northern Vietnam	Rural	Plains	No	15.4	Hb < 12 g/dL	9
Casey et al,^16^ 2017	2000-2005	A cross-sectional study	389	Random	Northern Vietnam	Rural	Mountains	Yes	37.8	Hb < 120 g/L	9
Berger et al,^17^ 2005	≤ 1999	Case-control study	864	Random	Northern Vietnam	Rural	Plains	No	45.6	Hb < 120 g/L	9

^a^Hemoglobin; ^b^Grams per liter; ^c^Grams per deciliter.

###  Pooled meta-analysis

**Figure 2:**
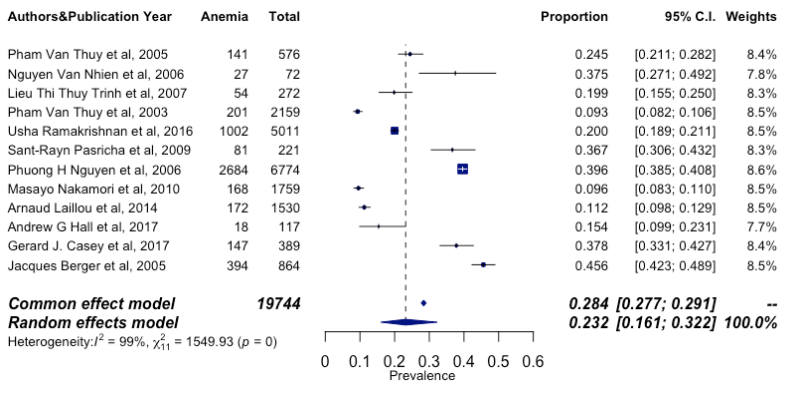
Pooled anaemia percentage among 19,744 non-pregnant women in Vietnam

**Figure 3:**
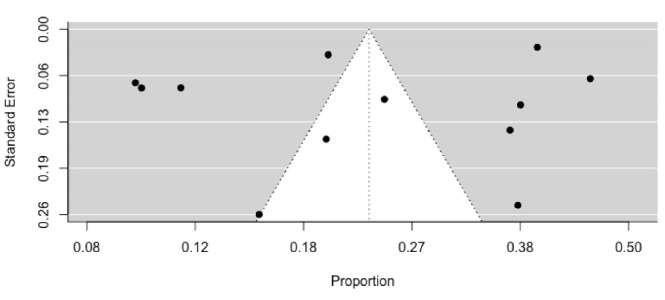
Funnel plot of the prevalence of anaemia among non-pregnant Vietnamese women

 In a total sample of 19 744 non-pregnant women, the pooled prevalence of anaemia was 23.2% (Q = 1549.93; df = 11; *P*< 0.001; 95% CI: 16.1-32.2, I^2^ = 99.29%, based on 12 studies). Because there was statistically significant heterogeneity among the included publications, we utilized a random effect meta-analysis approach to estimate the pooled prevalence of anaemia among non-pregnant Vietnamese women. Figure 2 depicts a forest plot of the prevalence estimates with their associated CIs. In the sensitivity analysis, no single included study significantly impacted the overall pooled prevalence of anaemia, indicating the remarkable consistency of the finding (Table 2). The funnel plot, shown in Figure 3, revealed that there was almost no publication bias, and the results of the Egger regression test (*P* = 0.9081) strongly supported this conclusion.

**Table 2:** One-study removed sensitivity analysis of the pooled percentage of anaemia among non-pregnant women of reproductive age in Vietnam

**The omitted study**	**Pooled percentages**	**I** ^ 2 ^ ** [95% CI]**	**Q**	***P***	**Egger regression test **
1	0.2307 [0.1556; 0.3280]	99.4% [99.2%; 99.5%]	1545.51	< 0.01	*P* = 0.2661
2	0.2216 [0.1505; 0.3140]	99.4% [99.2%; 99.5%]	1547.01	< 0.01	*P* = 0.1994
3	0.2351 [0.1600; 0.3313]	99.4% [99.2%; 99.5%]	1540.27	< 0.01	*P* = 0.2706
4	0.2502 [0.1770; 0.3410]	99.2% [99.0%; 99.3%]	1198.93	< 0.0001	*P* = 0.3256
5	0.2351 [0.1515; 0.3459]	99.2% [99.1%; 99.4%]	1328.31	< 0.0001	*P* = 0.1324
6	0.2215 [0.1498; 0.3149]	99.4% [99.2%; 99.5%]	1542.46	< 0.01	*P* = 0.2056
7	0.2191 [0.1525; 0.3043]	98.7% [98.3%; 99.0%]	771.2	< 0.0001	*P* = 0.7724
8	0.2496 [0.1753; 0.3424]	99.2% [99.0%; 99.4%]	1272.02	< 0.0001	*P* = 0.3414
9	0.2465 [0.1711; 0.3413]	99.3% [99.1%; 99.4%]	1342.21	< 0.0001	*P* = 0.3337
10	0.2395 [0.1640; 0.3358]	99.4% [99.2%; 99.5%]	1540.65	< 0.01	*P* = 0.2639
11	0.2206 [0.1487; 0.3146]	99.3% [99.2%; 99.5%]	1532.77	< 0.0001	*P* = 0.2053
12	0.2154 [0.1451; 0.3076]	99.3% [99.1%; 99.4%]	1421.55	< 0.0001	*P* = 0.1798

 After recognizing the studies’ heterogeneity, subgroup analysis was performed based on several characteristics. As Table 3 summarizes, analyses by study design revealed that case-control studies indicated a slightly higher significance in the pooled prevalence of anaemia than cross-sectional studies (25.4%; 95% CI: 16.0-37.9 vs 21.9%; 95%: 14.9-31.1). The prevalence of anaemia was more common in studies that used a non-random sample method (37.5%; 95% CI: 7.1-49.2) than in studies that used a random sampling method (22.1%; 95% CI:16.2-29.5). Regarding the ethnic minority demographic, studies that included ethnic minorities in some or all of their participants had a significantly higher prevalence of anaemia (25.1%; 95% CI: 14.7-39.4) than studies that did not include individuals from an ethnic minority (22.5%, 95% CI: 15.8-31.1).

**Table 3:** Subgroup analysis of studies included in meta-analysis on the prevalence of anaemia among non-pregnant women of reproductive age in Vietnam

**Variables**	**Characteristics**	**Number of studies**	**Number of participants**	**Prevalance with 95%**	**I-squared statistic**	***P*** ** value**
By design	Case-control study	4	6568	25.4 (16.0-37.9)	99%	< 0.01
	Cross-sectional study	6	4872	21.9 (14.9-31.1)	99%	< 0.01
By sampling	Random	9	11368	22.1 (16.2-29.5)	99%	< 0.01
	Non-random	1	71	37.5 (27.1-49.2)	-	-
By periods	Before 2000	2	7638	42.6 (25.0-62.3)	91%	< 0.01
	2000-2005	5	3468	23.5 (15.4-34.1)	98%	< 0.01
	After 2005	5	8638	16.9 (10.8-25.4)	98%	< 0.01
By ethnic minority	Yes	3	5672	25.1 (14.7-39.4)	97%	< 0.01
	No	7	5768	22.5 (15.8-31.1)	99%	< 0.01
By geographics	Plain	6	5547	20.6 (13.9-29.5)	99%	< 0.01
	Mountain	4	5893	27.7 (17.7-40.5)	97%	< 0.01
By regions	Northern Vietnam	9	11168	23.7 (17.4-31.4)	99%	< 0.01
	Central Vietnam	1	272	19.9 (15.5-25.0)	-	-
By areas	Rural	8	7522	28.7 (21.0-37.8)	98%	< 0.01
	Urban	2	3918	9.4 (4.4-18.9)	0	= 0.8

 In terms of geographics, regions, and areas, the prevalence of anaemia in Vietnam women was shown to be relatively greater in studies that were carried out in the mountains, in Northern Vietnam, and in rural areas (27.7%; 95% CI: 17.7-40.5, 23.7%; 95% CI: 17.4-31.4 and 28.7%; 95% CI: 21.0-37.8, respectively) than in studies conducted in the plains, in Central Vietnam, and in urban areas of Vietnam (20.6%; 95% CI: 13.9-29.5, 19.9%; 95% CI: 15.5-25.0, 9.4%; 95% CI: 4.4-18.9, respectively).

**Figure 4:**
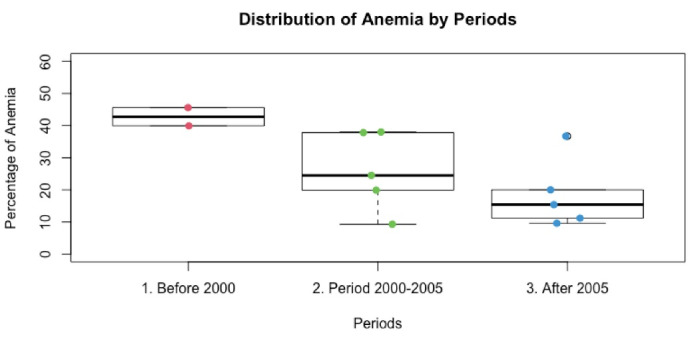
A box plot depicting the decreasing prevalence of anaemia among non-pregnant Vietnamese women over roughly two decades

 Anaemia prevalence was stratified by collecting periods: before 2000, 2000-2005, and after 2005 (Figure 4**)**. The prevalence of anaemia was 42.6% (25.0-62.3), 23.5% (15.4-34.1) and 16.9% (10.8-25.4), accordingly to the times during which collections were made. From 1995 to 2013, the pool prevalence of anaemia decreased from 42.6% to 16.9%, a significant reduction.

## Discussion

 In this comprehensive systematic review, which included a meta-analysis of cross-sectional and case-control surveys carried out in Vietnam over the past two decades and involving 19,744 non-pregnant women of reproductive age, it was possible to arrive at accurate estimates of the prevalence of anaemia in this population. With the WHO anaemia cut point of 12.00 g/dL for non-pregnant women, the findings of this meta-analysis encompassing 12 articles revealed that the prevalence of anaemia in this demographic in Vietnam was 23.2% (95% CI: 16.1-32.2). This review also revealed a nearly two-decade-long decline in the prevalence of anaemia among women in Vietnam. According to the WHO categorization of public health significance of anaemia in communities, the percentage of anaemia in women of reproductive age in Vietnam is at the second most hazardous level (Moderate).^26^

 Compared to the findings from other countries in seven South and Southeast Asian nations,^27^ our finding was significant lower (23.2% vs 52.6%). The differences in diet, geography, and culture between Vietnam and these nations might be a probable explanation for the disparity in anaemia.^28^ The first national nutrition plan to be formally recognized in Vietnam was ratified by the Prime Minister in 1995 and titled the National Plan of Action for Nutrition 1995-2000. Several interventions were then in place in Vietnam to fight anaemia in reproductive-age women using integrated, multifactorial, and multisectoral approaches, which recognizes the complexities of anaemia and may lead to beneficial methods. The execution of the National Plan of Action for Nutrition (1995-2000), advocacy and other communication initiatives to boost food production and consumption in Vietnam during the last decade has been effective. The recent decade has seen an increase in the production of the most important foods. Dietary intake has increased significantly compared to 1987, with more people consuming higher-quality meals, including meat, fish, fat oils, and ripe fruits.^29^ The National Nutrition Strategies for 2001-2010 and 2011-2020 were developed as the following official document controlling nutrition policy in Vietnam as a follow-up to the first National Plan, which served as the official document regulating nutrition policy in Vietnam at the time. The nutrition status of the Vietnamese people, in general, has significantly improved after more than twenty years of implementing these National Plans with comprehensive approaches, multi-sector cooperation, and government guidance at different levels. This improvement coincides with increasing awareness of nutrition issues among the Vietnamese.^30^ Notably, even though many parts of Vietnam have seen general improvements in lowering the prevalence of anaemia, our findings highlight persistent disparities across mountains, in Northern Vietnam, in rural areas and ethnic minority groups that have either remained unchanged or fallen behind the general improvements of other locations within the country. We presume that our results are consistent with the result of Harris et al^31^ that although Vietnam has effectively decreased population stunting, ethnic minority groups are being systematically left behind, hindering progress toward national reductions.^31,32^ The rationale suggested for this predicament is that government policies and initiatives may not have been culturally adaptive and responsive, and there have been allegations of negative attitudes and prejudice by health workers toward minorities.^33^ In addition, a significant portion of Vietnam’s officially recognized ethnic minority groups is finding that they are unable to properly interact with the programs developed for their respective areas by the central government.^31^

 There are, however, further significant gaps in the research about the prevalence of non-pregnant Vietnamese women that need to be addressed. First, when the data were pooled, a substantial amount of heterogeneity was discovered. One possible explanation for this heterogeneity is that the studies that were identified had distinct features and unexamined factors. Second, because the data were only available for research carried out in Northern and Central Vietnam, the ability to generalize the findings to Southern Vietnam may be restricted. Third, since meta-analysis by its very nature utilizes aggregated group data, other confounding factors that may have an influence on the prevalence of anaemia were not considered and excluded, and this may have caused the estimate to be inaccurate. Fourth, we regret that due to limited access to subscription databases at developing country universities, we were unable to study any of the other databases, which may have resulted in publication bias. Strikingly, our findings reflect the best estimations that are presently available and might serve as a point of reference for further designing policies pertaining to public health. We conclude that our searches of the PubMed and Scopus databases were sufficient since our research objective is limited to studies on the anaemia percentage. Finally, due to our research primarily focused on the general population, the pooled estimate for the prevalence of anaemia among Vietnamese women may not accurately reflect the extent of the problem in other specialized populations.

## Conclusion

 To our knowledge, this was the first systematic study to assess the pooled prevalence of anaemia among non-pregnant women of reproductive age in Vietnam during the past two decades. This population in Vietnam is showing a decreasing trend in the prevalence of anaemia as a result of our comprehensive research. The data also highlights disparities in geographical and ethnic minority groups in the prevalence of anaemia among non-pregnant women of reproductive age. The larger proportion is seen in the highlands, Northern Vietnam and rural areas. Efforts to combat ethnic health disparities should concentrate on high-risk groups by establishing control and preventative techniques that address anaemia’s socio-cultural and dietary specificities in indigenous populations.

## Author Contributions

**Conceptualization:** Nguyen Trung Kien and Tran Quang Duc.

**Data curation: **Tran Quang Duc.

**Formal Analysis: **Nguyen Trung Kien and Tran Quang Duc.

**Funding acquisition: **None.

**Investigation: **Bui Thi Thanh Tuyen and Dinh Thi Phuong Hoa.

**Methodology: **Nguyen Trung Kien and Tran Quang Duc.

**Project administration:** Vu Thi Quynh Chi and Bui Thi Thanh Tuyen

**Resources:** Tran Quang Duc.

**Software:** Tran Quang Duc and Phan Ngoc Quang.

**Supervision: **Nguyen Trung Kien.

**Validation:** Nguyen Trung Kien, Tran Quang Duc and Phan Ngoc Quang.

**Visualization: **Tran Quang Duc and Vu Thi Quynh Chi.

**Writing – original draft:** Tran Quang Duc and Bui Thi Thanh Tuyen.

**Writing – review & editing:** Vu Thi Quynh Chi and Phan Ngoc Quang.

## Funding

 Nil.

## Ethical Approval

 Not required.

## Competing Interests

 There are no conflicts of interest.
